# Identifying and prioritizing recommendations to optimize transitions across the care journey for hip fractures: Results from a mixed-methods concept mapping study

**DOI:** 10.1371/journal.pone.0307769

**Published:** 2024-08-26

**Authors:** Sara J. T. Guilcher, Lauren Cadel, Amanda C. Everall, Susan E. Bronskill, Walter P. Wodchis, Kednapa Thavorn, Kerry Kuluski

**Affiliations:** 1 Leslie Dan Faculty of Pharmacy, University of Toronto, Toronto, Ontario, Canada; 2 Institute of Health Policy, Management and Evaluation, University of Toronto, Toronto, Ontario, Canada; 3 ICES, Toronto, Ontario, Canada; 4 Institute for Better Health, Trillium Health Partners, Toronto, Ontario, Canada; 5 Ottawa Hospital Research Institute, University of Ottawa, Ottawa, Ontario, Canada; 6 School of Epidemiology and Public Health, University of Ottawa, Ottawa, Ontario, Canada; University of Campania Luigi Vanvitelli: Universita degli Studi della Campania Luigi Vanvitelli, ITALY

## Abstract

**Background:**

Individuals who experience a hip fracture have numerous care transitions. Improving the transition process is important for ensuring quality care; however, little is known about the priorities of different key interest groups. Our aim was to gather recommendations from these groups regarding care transitions for hip fracture.

**Methods:**

We conducted a concept mapping study, inviting persons with lived experience (PWLE) who had a hip fracture, care partners, healthcare providers, and decision-makers to share their thoughts about ‘what is needed to improve care transitions for hip fracture’. Individuals were subsequently asked to sort the generated statements into conceptual piles, and then rate by importance and priority using a five-point scale. Participants decided on the final map, rearranged statements, and assigned a name to each conceptual cluster.

**Results:**

A total of 35 participants took part in this concept mapping study, with some individuals participating in multiple steps. Participants included 22 healthcare providers, 7 care partners, 4 decision-makers, and 2 PWLE. The final map selected by participants was an 8-cluster map, with the following cluster labels: (1) access to inpatient services and supports across the care continuum (13 statements); (2) informed and collaborative discharge planning (13 statements); (3) access to transitional and outpatient services (3 statements); (4) communication, education and knowledge acquisition (9 statements); (5) support for care partners (2 statements); (6) person-centred care (13 statements); (7) physical, social, and cognitive activities and supports (13 statements); and (8) provider knowledge, skills, roles and behaviours (8 statements).

**Conclusions:**

Our study findings highlight the importance of person-centred care, with active involvement of PWLE and their care partners throughout the care journey. Many participant statements included specific ideas related to continuity of care, and clinical knowledge and skills. This study provides insights for future interventions and quality improvement initiatives for enhancing transitions in care among hip fracture populations.

## Introduction

Hip fractures are one of the most common reasons for fall-related hospitalizations in adults over the age of 65 [1, 2]. Older adults with hip fractures often experience functional decline and on-going morbidity after their injury [3–6]. Risk factors for hip fracture may include history of osteoporosis, falls, physical inactivity, medication use (cimetidine, psychotropic anxiolytics/ hypnotics, barbiturates, opioids, antihypertensives, benzodiazepines, anticonvulsants, sedatives, caffeine, and antidepressants), chronic health conditions, older age, and being female [7–9]. Unfortunately, approximately 13.5% of persons die within six months of sustaining a hip fracture injury [10]. Of those that survive, only 40% of persons can perform activities of daily living independently following the first year post-fracture and persons often have a decline in health-related quality of life [11–14].

Smooth care transitions can be challenging for this population, as individuals who experience a hip fracture undergo an average of 3.5 transitions between care settings in the first six months following a hip fracture [15]. These transitions occur within and between many different healthcare providers and diverse settings including hospitals, inpatient rehabilitation units, outpatient clinics, home care, assisted living facilities, and long-term care homes [15–19]. Importantly, some care transitions are a key part of the hip fracture journey and standards of care [20, 21]. For example, rehabilitation post-hip fracture can improve the physical and social aspects of reintegration into the community [21]. Despite the importance and need for some of these transitions, care transitions that result in increased hospital readmissions and emergency department visits, adverse medication events, and overall poor patient and caregiver experience and satisfaction are considered to be suboptimal [22–25]. The one year direct attributable healthcare costs for hip fractures in Canada is $1.1 billion [26] and is estimated to increase to $2.4 billion by 2041 in part due to an aging population [27].

Most research on care transitions in the general population, as well as hip fracture more specifically, has focused on the acute transition from hospital to home rather than across the care continuum [28]. In efforts to address this gap for hip fracture, Stolee and colleagues synthesized multi-site ethnographic qualitative research, which involved perspectives from patients, care partners and clinicians [28]. They proposed a framework that outlines potential areas of intervention. The six broad domains proposed include the following: patient involvement and choice (patients and caregivers are educated on options and able to make decisions), role of family care partners (family caregivers provide information to healthcare team, provide care, and want to be actively involved in care decisions), relationships (relationships between healthcare providers, and between patients and family caregivers), role coordination (understanding of healthcare providers’ roles within and across settings), documentation (written communication about patient’s health and status), and information sharing (use of written and verbal communication to share information formally and informally). These domains align with previous research conducted by this team, which highlighted the need to focus on improved communication and information sharing among patients, their care partners, and health professional team [16–18].

Similarly, our team has conducted several studies exploring experiences with hip fracture along the care continuum in Ontario, Canada [25, 29, 30]. Communication was also identified as an important area of focus, as care partners and healthcare providers often had differing expectations on the roles of care partners, providers, and the health system more broadly [30]. Building on this work, Cadel and colleagues explored service recommendations from the perspectives of patients, care partners, healthcare providers, and decision-makers [29]. Three main categories of recommendations were outlined: (1) hospital-based recommendations; (2) community-based recommendations; and (3) cross-sectoral based recommendations. Hospital-based recommendations focused on improving relationships, communication, and staffing levels. Community-based recommendations included the early identification of at-risk individuals and providing preventative and educational programs. Cross-sectoral based recommendations were grounded in enhanced system navigation through communication and care navigators, particularly within primary and community care settings. While these recommendations from Stolee and Guilcher are informative as an initial step for broad areas of improvement, there is a need for more specific design ideas and priority setting to facilitate actionable implementation strategies for the development of implementable quality improvement initiatives and complex interventions [31, 32].

Further research is warranted with key interest group engagement to understand from their perspectives on specific strategies to assist in care transitions for hip fracture and more importantly, their perspectives on which areas are of a priority. This priority setting step is important to engage in prior to the development and implementation of an intervention to best understand the causal and contextual factors that can be modified [33]. Therefore, in using creative partnership design principles [34], the aim of this study was to create a list of actionable and prioritized recommendations to improve care transitions for individuals with hip fracture from the perspectives of persons with lived experience (PWLE), care partners, healthcare providers, and decision-makers.

## Methods

### Study design

A mixed methods study was conducted, following the concept mapping methodology outlined by Kane and Trochim [35]. Concept mapping uses a collaborative approach to data collection and analysis that is useful for gathering perspectives from a range of participants for planning and evaluation purposes [36, 37]. Given its action-oriented nature, concept mapping was ideal for the purposes of this project in developing actionable recommendations with creative partnership design principles [35, 38]. This structured methodology is comprised of six sequential steps, including: preparation, brainstorming, sorting and rating, analysis, mapping and interpretation, and utilization [35].

### Preparation

The preparation step consisted of two key tasks: identifying the focal prompt (research question) and recruiting participants. The focal prompt developed by the research team to guide all stages of this study was: *what do you think is needed to improve care transitions for hip fracture*?

### Participant inclusion criteria

Participants included persons who had experienced a hip fracture, unpaid care partners (e.g., family or friends), healthcare providers, and decision-makers. PWLE were required to be at least 50 years of age, at the time of the interview, to align with quality standards in Ontario. All other participants were required to be 18 years of age or older. All participants had to be able to speak English. PWLE were required to have received care for any type of hip fracture in Ontario (e.g., osteoporotic, traumatic); care partners were required to have provided care for an individual who experienced a hip fracture; healthcare providers and decision-makers were required to have provided care in a paid capacity for individuals with hip fracture or to influence policy/procedures relating to hip fracture.

### Sampling strategy

We used a multipronged purposive sampling strategy [39, 40]. Participants were selected for variation in geographical setting (rural, urban). Healthcare providers and decision-makers were also selected for variation in profession and health sector. To recruit participants, study flyers were posted in healthcare facilities, on partnering professional and healthcare organizations websites, and on social media. Most PWLE and care partners were recruited by healthcare providers in two partnering acute care hospitals (one urban, one rural) using a consent to contact method whereby a healthcare provider identified eligible individuals, gave them a brief description of the study, and obtained verbal or written consent for a member of the research team to contact them. Healthcare providers and decision-makers were identified through the research teams’ contacts in the two partnering hospitals, as well as through health professional organizations and associations. All recruitment occurred between April 2022 and June 2023.

### Idea generation: Brainstorming

Brainstorming sessions were guided by the focal prompt: *what do you think is needed to improve care transitions for hip fracture*? Sessions were held both synchronously and asynchronously, depending on participant availability. Synchronous sessions were conducted as focus groups or interviews and were facilitated by trained members of the research team (ACE, MSc; LC, MSc, PhD candidate). Focus groups were conducted virtually via Zoom and interviews were conducted by Zoom or by telephone between July 2022 and September 2022. The focus groups and interviews followed a semi-structured brainstorming interview guide and allowed for group discussion to generate additional ideas related to the focal prompt. Participants were divided into two groups: a) PWLE and care partners, and b) healthcare providers and decision-makers, to maximize comfort in sharing their thoughts and experiences. Asynchronous sessions were conducted independently by participants using the online concept mapping platform, groupwisdom™, and were only made available to those individuals who were unable to attend a synchronous session. All participants completed a short, online, demographic survey prior to participation.

Statements from all brainstorming sessions, across all participant types, were combined into an Excel spreadsheet for statement synthesis. The original list of brainstorming items needed to be reduced to a manageable number (e.g., less than 100) for the sorting and rating phase [41]. Therefore, two members of the research team (ACE, LC) initially reviewed each of the statements to de-duplicate and remove items that did not answer the focal prompt. The research team then engaged in the final rounds of condensing (combining similar ideas) and rewording the statements for consistency and clarity. The final statement list was added to groupwisdom™ for the subsequent steps of concept mapping.

### Sorting and rating

The sorting and rating activities were completed independently by participants on groupwisdom™. Participants created groups of statements based on their perceived conceptual similarity and labelled each group. Participants then rated each statement on two dimensions–importance and priority of the recommendation. For both rating questions, a five-point Likert-type scale was used (importance: 1 = not at all important, 2 = slightly important, 3 = moderately important, 4 = very important, 5 = extremely important; priority: 1 = not at all a priority, 2 = slight priority, 3 = moderate priority, 4 = high priority, 5 = extreme priority).

### Concept mapping analysis

All analyses were conducted using groupwisdom™. A point map was created (see S1 Fig), which is a visual representation of all the statements, with the relative distance between the points representing how frequently the statements were sorted together by participants [35]. The goodness of fit is represented by a stress value, with an adequate stress value for concept mapping projects being between 0.205 and 0.365 [35]. Cluster map solutions were created through hierarchical cluster analysis, which groups statements (creates boundaries around statements) based on their relative distance. Cluster map solutions were reviewed by the research team and through group discussion, two cluster solutions were chosen to present to participants. The starting point in reviewing the cluster map solutions was based on the average number of clusters created by participants in the sorting activity. The research team then sequentially reviewed the cluster maps to identify those with conceptually distinct clusters. For each cluster, statement bridging values were averaged to better understand how the statements within each cluster fit together. Bridging values range from 0.00 to 1.00, with a value of 0 indicating that participants sorted statements similarly and 1.00 statements were sorted differently. Clusters with higher bridging values have statements that are likely interconnected with other clusters and interrelated.

### Mapping and interpretation session

The synchronous mapping session was held in June 2023 with a subset of participants from the previous phases. It was conducted virtually on Zoom and was led by a trained member of the research team (SJTG, PhD). The research team presented the participants with two cluster map solutions (7-cluster and 8-cluster; see S2 Fig), which had been previously selected as two solutions that best represented the data. Through group discussion guided by the facilitator, participants reached consensus on the final cluster map solution. The statements within each cluster were then reviewed and participants were able to move statements between clusters to ensure conceptual similarity of all statements contained within each cluster. Participants suggested labels for the clusters, which were finalized by the research team following the session.

Using groupwisdom™, the research team created additional visual representations of the data. Cluster rating maps were created for importance and priority to visually display which clusters had a relatively higher average rating. Pattern match diagrams were created to compare average cluster ratings based on different participant demographic variables. Lastly, a go-zone diagram was created to display the mean statement values for importance and priority. The go-zone is the upper right quadrant of the diagram, which displays the statements that were rated higher than average on both dimensions.

### Utilization and knowledge mobilization

The research team will engage in webinars and meetings with key stakeholders to inform the potential implementation of the actionable recommendations to improve transitions in care for adults with hip fracture.

### Ethics

This study received ethics approval from the Research Ethics Boards of the University of Toronto (#35779) and two acute hospitals (#18–047 and #893) in different health regions in Ontario, Canada. All participants provided written or verbal consent prior to participation.

## Results

### Participant demographics

A total of 35 participants took part in this concept mapping study (see Table 1 for participant demographics), with some of those individuals participating in multiple steps; 32 participated in brainstorming, 23 completed sorting and rating, and 11 took part in mapping. Across the three steps of concept mapping (brainstorming, sorting/rating, and mapping), most participants were healthcare providers (n = 21, 66%; n = 15, 65%; n = 6, 55%; respectively) and female, who also identified as women (n = 25, 78%; n = 17, 74%; n = 10, 91%; respectively). Participants were mostly between 18–69 years of age (n = 32, 100%; n = 22, 96%; n = 10, 91%; respectively), with a relatively similar distribution across these age groups. Most participants identified as being white (n = 20; 63%, n = 14; 61%, n = 8; 73%; respectively), with limited representation across other races.

**Table 1:** Participant numbers and demographics across the three steps of concept mapping.

Demographics	Brainstorming (n = 32)	Sorting and Rating (n = 23)	Mapping (n = 11)
***Participant Type***			
Persons with lived experience	2 (6%)	0 (0%)	0 (0%)
Care partners	5 (16%)	5 (22%)	3 (27%)
Healthcare providers	21 (66%)	15 (65%)	6 (55%)
Decision-makers	4 (13%)	3 (13%)	2 (18%)
***Sex***			
Male	7 (22%)	6 (26%)	1 (9%)
Female	25 (78%)	17 (74%)	10 (91%)
***Gender***			
Man	7 (22%)	6 (26%)	1 (9%)
Woman	25 (78%)	17 (74%)	10 (91%)
Other*	0 (0%)	0 (0%)	0 (0%)
***Age***			
18–39	11 (34%)	8 (35%)	3 (27%)
40–59	15 (47%)	11 (48%)	5 (45%)
60+	6 (19%)	4 (17%)	3 (27%)
***Race***			
Black	2 (6%)	1 (4%)	0 (0%)
East/ Southeast Asian	7 (22%)	5 (22%)	2 (18%)
Middle Eastern or South Asian	2 (6%)	3 (13%)	1 (9%)
White	20 (63%)	14 (61%)	8 (73%)
Other	1 (3%)	0 (0%)	0 (0%)

*Other: transgender, non-binary, other

### Cluster map

**Fig 1:**
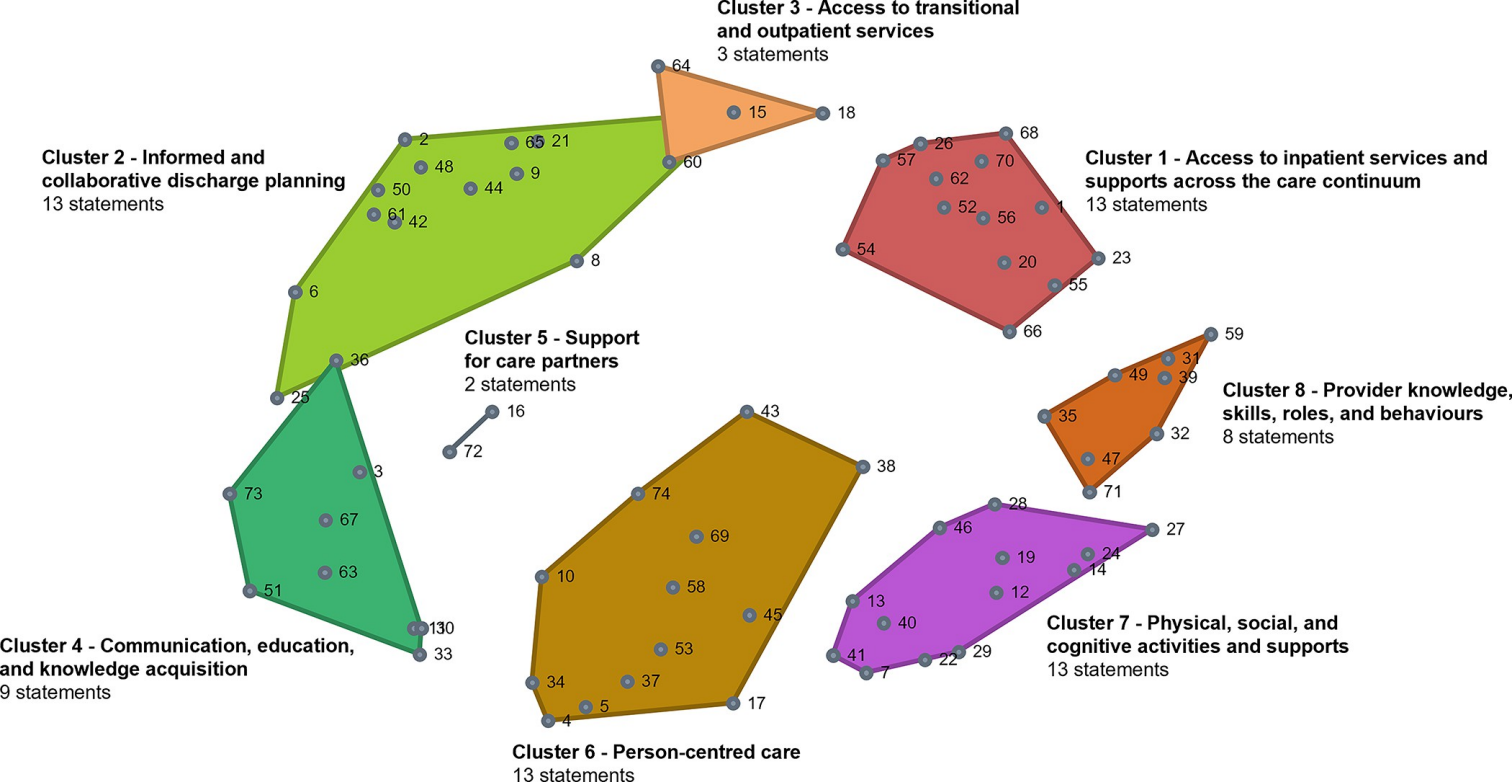
8-cluster map solution. Each shape represents a cluster containing statements (represented by dots) that were grouped together by participants. Each cluster has a title reflecting the overall concept of the statements assigned.

A total of 887 statements were generated by participants in the brainstorming sessions, with the synthesized list containing 74 unique statements. The stress value for the point map was 0.265 indicating a good fit between the multidimensional scaling input and the cluster configurations. The final cluster map selected by participants was the 8-cluster map (see Fig 1), with the following labels: (1) access to inpatient services and supports across the care continuum; (2) informed and collaborative discharge planning; (3) access to transitional and outpatient services; (4) communication, education and knowledge acquisition; (5) support for care partners; (6) person-centred care; (7) physical, social, and cognitive activities and supports; and (8) provider knowledge, skills, roles and behaviours. See Table 2 for cluster names, statements, and average statement ratings.

**Table 2:** Cluster names, statements, and average statement ratings.

Cluster Name	Statements	Mean Importance Rating	Meaning Importance Rating of Cluster	Mean Priority Rating	Mean Priority Rating of Cluster
CLUSTER 1—Access to inpatient services and supports across the care continuum Bridging value = 0.21	1—Increasing access to different types of rehabilitation services based on patients’ needs	4.30	4.15	4.09	3.99
	20—Having access to different healthcare providers (e.g., geriatricians, therapists, social workers)	4.43		4.30	
	23—Increasing staffing so providers can spend more time with patients	4.48		4.26	
	26—Having established partnerships between hospitals, rehabilitation facilities, and homecare services for consistent transitions	4.43		4.39	
	52—Reducing wait times for services or procedures	4.22		4.09	
	54—Ongoing follow-up with surgeon to assess recovery	4.00		3.74	
	55—Increasing physical therapy and exercise in all care settings, including on weekends	4.26		4.00	
	56—Having standardized care across care settings with the flexibility to customize based on patient needs and preferences	4.30		3.87	
	57—Avoiding unnecessary transitions, especially for those with cognitive impairment	4.22		4.04	
	62—Improving access to recreational therapy and group activities	3.70		3.61	
	66—Prioritizing care of patients who are most likely to decline	3.65		3.74	
	68—Improving the transfer of patients’ medical information between organizations and providers	4.17		4.04	
	70—Changing admittance requirements for rehabilitation facilities to permit those with cognitive impairment	3.78		3.70	
CLUSTER 2—Informed and collaborative discharge planning Bridging value = 0.45	2—Ensuring support (paid or unpaid) to meet patient needs when returning home	4.39	3.88	4.09	3.72
	8—Ensuring patients have one person who helps them coordinate their care transitions	3.43		3.52	
	9—Conducting home safety assessments prior to discharge	3.57		3.26	
	21—Including rehabilitation therapists’ recommendations when considering discharge dates and location	4.30		4.26	
	42—Setting discharge dates well in advance	3.83		3.57	
	44—Ensuring patients have home visits before discharge (e.g., day/weekend passes)	2.91		2.78	
	48—Having discharge requirements based on patients’ abilities to perform daily tasks	3.91		4.00	
	50—Understanding patients’ abilities to perform daily activities after discharge (e.g., drive, cook, dress)	4.04		3.96	
	61—Including home and community care providers in discharge planning	4.26		4.09	
	65—Avoiding discharges on Fridays and weekends to minimize gaps in services or care	3.57		3.43	
	15—Having access to transitional spaces for those not yet ready to return home or waiting for a bed at their next point of care	4.22		4.13	
	6—Having early discussions about the possibility of long-term care placements	3.70		3.13	
	25—Patients and care partners being informed of what assistive devices are needed and where to get them	4.30		4.09	
CLUSTER 3—Access to transitional and outpatient services Bridging value = 0.43	18—Ensuring physical assistance with transfers between facilities (e.g., ambulance, assistance with getting into/out of car)	3.83	4.19	3.70	4.04
	60—Having access to necessary services and equipment regardless of ability to pay for it	4.35		4.17	
	64—Ensuring timely, consistent, and sufficient homecare services	4.39		4.26	
CLUSTER 4—Communication, education and knowledge acquisition Bridging value = 0.67	3—Routinely collecting information on patients’ abilities before hip fracture	3.87	4.07	3.39	3.86
	11—Having open conversations about what to expect about care and transitions	4.13		4.04	
	30—Patients and care partners knowing what questions to ask providers	3.78		3.70	
	33—Providing patients and care partners with ongoing communication throughout their care transitions	4.52		4.26	
	51—Accepting that recovery from hip fracture takes time	4.13		3.61	
	63—Managing expectations about service availability and accessibility	3.91		4.00	
	67—Addressing concerns and fears about going home	4.35		4.00	
	73—Knowing what services exist and how to access them (e.g., homecare, community, social)	4.26		4.26	
	36—Inquiring about patients’ finances to make appropriate referrals	3.70		3.48	
CLUSTER 5—Support for care partners Bridging value = 0.50	16—Ensuring care partners can provide in-person support for patients in all care settings	3.74	3.89	3.70	3.78
	72—Having more supports for care partners to address caregiver burden	4.04		3.87	
CLUSTER 6—Person-centred care Bridging value = 0.24	4—Respecting patients’ and care partners’ preferences, knowledge, and needs	4.35	4.15	4.09	3.95
	5—Ensuring patients and Care partners are included in decisions (e.g., goals, transition processes, and care plans)	4.61		4.35	
	10—Addressing concerns and fears about future falls	4.17		3.91	
	17—Bringing pieces of home (e.g., pictures, clothing) across the care transitions	2.96		2.74	
	34—Communicating with patients and care partners in their primary language	4.22		4.09	
	37—Ensuring patients’ and care partners’ questions are answered	4.48		4.26	
	38—Being informed about medication changes (e.g., new, adjustments)	4.09		3.91	
	43—Ensuring patients’ physical needs (e.g., eating, toileting) are met before transferring to a new facility	4.09		3.74	
	45—Discussing patients’ goals early and often throughout their care transitions	4.26		4.13	
	53—Using clear and simple language to improve patient and caregiver understanding	4.48		4.26	
	58—Having a positive relationship with healthcare providers	4.26		4.00	
	69—Tailoring how information is shared based on needs and preferences	3.96		3.78	
	74—Trusting that patients are receiving appropriate care that is in their best interest	4.09		4.13	
CLUSTER 7—Physical, social, and cognitive activities and supports Bridging value = 0.15	7—Informing patients about the rationale for each exercise as they relate to their goals	4.00	3.94	3.61	3.82
	12—Being compassionate while caring for patients	4.65		4.52	
	13—Having caregiver involvement with physical therapy	3.43		3.52	
	14—Ensuring social stimulation (e.g., conversation, recreational activities)	3.74		3.78	
	19—Using technology to share education materials about exercises and mobilization	3.04		3.00	
	22—Encouraging patient motivation with rehabilitation	4.35		4.35	
	24—Ensuring patients see the same providers at the same time throughout their stay	3.04		3.00	
	27—Addressing delirium and cognitive impairment through best practices (e.g., visual aids, social interactions, routines)	4.39		4.17	
	28—Improving the hospital environment to be more conducive to recovery (e.g., noise, light, social engagement, food)	3.83		3.57	
	29—Having individualized exercises that change over time to meet patient goals	4.39		4.04	
	40—Providing social, mental, and physical activities regardless of cognitive status	4.09		3.96	
	41—Ensuring patients feel supported physically and emotionally	4.39		4.22	
	46—Providers introducing themselves by name and role each time they interact with patients or care partners	3.91		3.87	
CLUSTER 8—Provider knowledge, skills, roles and behaviours Bridging value = 0.22	31—Ensuring providers use the proper transferring techniques (e.g., sit to stand)	4.43	4.32	4.30	4.14
	32—Conducting frequent patient medication reviews (e.g., adding, changing, removing medications)	4.13		3.78	
	35—Ongoing patient assessments (e.g., geriatric, cognitive, functional) to identify, monitor, and manage clinical conditions	4.35		4.22	
	39—Having a care team that works well together	4.52		4.35	
	47—Practicing everyday activities (e.g., dressing, feeding, stairs, toileting) during recovery	4.22		4.13	
	49—Routinely screening for delirium and cognitive impairment	4.04		3.83	
	59—Having the necessary knowledge and skills to provide rehabilitative care for patients who have cognitive impairment	4.35		4.22	
	71—Having adequate pain management	4.48		4.30	

### Cluster 1 –Access to inpatient services and supports across the care continuum

Cluster 1 contained 13 statements that related to access to supports and services both in inpatient acute care and across the care continuum (bridging value = 0.21). Within this cluster, the statement rated highest on importance was *23—increasing staffing so providers can spend more time with patients* (mean = 4.48), and the statement rated lowest on importance was *66—prioritizing care of patients who are most likely to decline* (mean = 3.65). The statement rated most highly on priority was *26—having established partnerships between hospitals*, *rehabilitation facilities*, *and homecare services for consistent transitions* (mean = 4.39), and the statement rated lowest on priority was *62—improving access to recreational therapy and group activities* (mean = 3.61).

### Cluster 2 –Informed and collaborative discharge planning

Cluster 2 contained 13 statements that related to discharge planning (bridging value = 0.45). The statement rated highest on importance was *2 –ensuring support (paid or unpaid) to meet patient needs when returning home* (mean = 4.39). The statement rated lowest on importance and priority was *44 –ensuring patients have home visits before discharge (e*.*g*., *day/weekend passes)* (mean = 2.91, 2.78; respectively). The statement rated highest on priority was *21 –including rehabilitation therapists’ recommendations when considering discharge dates and location* (mean = 4.26).

### Cluster 3 –Access to transitional and outpatient services

Cluster 3 contained three statements that related to the access of transitional and outpatient services (bridging value = 0.43). The statement rated highest on importance and priority was *64—ensuring timely*, *consistent*, *and sufficient homecare services* (mean = 4.39, 4.26; respectively), while *18—ensuring physical assistance with transfers between facilities (e*.*g*., *ambulance*, *assistance with getting into/out of car)* was rated lowest on both importance and priority (mean = 3.83, 3.70; respectively).

### Cluster 4 –Communication, education, and knowledge acquisition

Cluster 4 contained nine statements that related to open and ongoing communication, education, and knowledge acquisition for patients, care partners, and healthcare providers (bridging value = 0.67). The statement rated highest on importance and priority was *33—providing patients and care partners with ongoing communication throughout their care transitions* (mean = 4.52, 4.26; respectively). Another statement that was rated most highly on priority was *73—knowing what services exist and how to access them* (e.g., homecare, community, social) (mean = 4.26). The statement rated lowest on importance was *36—inquiring about patients’ finances to make appropriate referrals* (mean = 3.7), while *3—routinely collecting information on patients’ abilities before hip fracture* (mean = 3.39) was rated lowest on priority.

### Cluster 5 –Support for care partners

Cluster 5 contained two statements that were specific to supports provided or received by care partners (bridging value = 0.50). The statement rated highest on both importance and priority was *72—having more supports for care partners to address caregiver burden* (mean = 4.04, 3.87; respectively). The statement rated lowest on both importance and priority was *16—ensuring care partners can provide in-person support for patients in all care settings* (mean = 3.74, 3.70; respectively).

### Cluster 6 –Person-centred care

Cluster 6 contained 13 statements that related to providing person-centred care (bridging value = 0.24). The statement rated highest on importance and priority (mean = 4.61, 4.35) was *5—ensuring patients and care partners are included in decisions (e*.*g*., *goals*, *transition processes*, *and care plans)*, while *17—bringing pieces of home (e*.*g*., *pictures*, *clothing) across the care transitions* was rated lowest on both importance and priority (mean = 2.96, 2.74; respectively).

### Cluster 7 –Physical, social, and cognitive activities and supports

Cluster 7 contained 13 statements that related to providing care and supports aligning with individual’s physical, social, and cognitive well-being (bridging value = 0.15). The statement rated highest on both importance and priority was *12—Being compassionate while caring for patients* (mean = 4.65, 4.52; respectively). Two statements were rated lowest on importance, with the latter also being rated lowest on priority *19—Using technology to share education materials about exercises and mobilization* (mean = 3.04) and *24—Ensuring patients see the same providers at the same time throughout their stay* (mean = 3.04, 3.00; respectively).

### Cluster 8 –Provider knowledge, skills, roles, and behaviours

Cluster 8 contained eight statements that related to the knowledge, behaviours, skills, and roles of healthcare providers involved in care transitions (bridging value = 0.22). Statement 39—*having a care team that works well together* was rated most highly on both importance and priority (mean = 4.52, 4.35; respectively). The statement rated lowest on importance was *49—Routinely screening for delirium and cognitive impairment* (mean = 4.04), and the statement rated lowest on priority was *32—Conducting frequent patient medication reviews (e*.*g*., *adding*, *changing*, *removing medications)* (mean = 3.78).

### Cluster rating maps

Overall, statements were rated relatively high on both importance and priority with all clusters having a mean rating of greater than three (moderate importance/ priority; see Table 2, S3 and S4 Figs). Participants rated Cluster 8—*provider knowledge*, *skills*, *roles*, *and behaviours* as the most important (mean = 4.32) and highest priority cluster (mean = 4.14). The next most important clusters, as rated by participants, were Cluster 3—*access to transitional and outpatient services* (mean = 4.19), Cluster 1—*access to inpatient services and supports across the care continuum* (mean = 4.15), and Cluster 6—*person-centred care* (mean = 4.15). Similarly, Cluster 3—*access to transitional and outpatient services* (mean = 4.04) and Cluster 1—*access to inpatient services and supports across the care continuum* (mean = 3.99) were the clusters rated as the next highest priorities. Participants rated Cluster 2—*informed and collaborative discharge planning* as the least important (mean = 3.88) and lowest priority cluster (mean = 3.72); however still rated relatively high as both were above neutral.

### Go-zone diagram: Identifying the top statements based on importance and priority correlation

**Fig 2:**
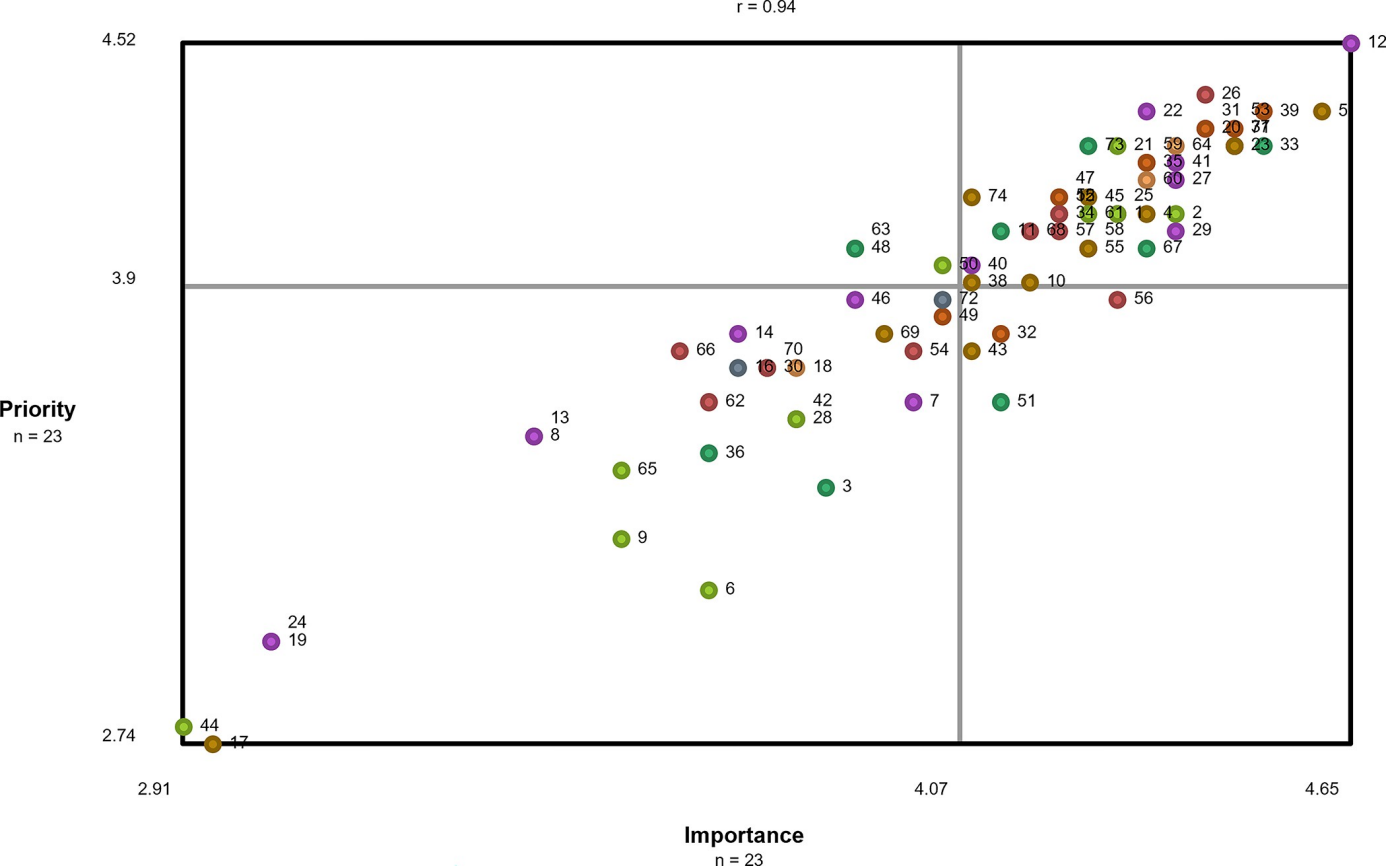
Go-zone diagram. Each dot corresponds to a statement. The colour of the dot corresponds to the cluster it is contained within (see Fig 1). The go-zone is the upper, right quadrant and displays statements that participants rated above average on both importance and priority.

A strong correlation between importance and priority was identified in the go-zone diagram (r = 0.94), which can be seen in Fig 2. Statements that were rated highly on importance were typically rated highly on priority as well. Of the 74 statements, over half (n = 41, 55%) were in the go-zone, which represents the statements that were rated above average on both importance and priority. Table 3 outlines all statements contained within the go-zone, organized by cluster, with the statements in descending order based on their overall average rating on importance and priority.

**Table 3:** Go-zone statements reflecting the top-rated statements on importance and priority.

Go Zone Statements	Mean Importance Rating	Mean Priority Rating	Overall Average Rating
**CLUSTER 1- Access to inpatient services and supports across the care continuum**			
26—Having established partnerships between hospitals, rehabilitation facilities, and homecare services for consistent transitions	4.43	4.39	4.41
23—Increasing staffing so providers can spend more time with patients	4.48	4.26	4.37
20—Having access to different healthcare providers (e.g., geriatricians, therapists, social workers)	4.43	4.30	4.37
1—Increasing access to different types of rehabilitation services based on patients’ needs	4.30	4.09	4.20
52—Reducing wait times for services or procedures	4.22	4.09	4.16
55—Increasing physical therapy and exercise in all care settings, including on weekends	4.26	4.00	4.13
57—Avoiding unnecessary transitions, especially for those with cognitive impairment	4.22	4.04	4.13
68—Improving the transfer of patients’ medical information between organizations and providers	4.17	4.04	4.11
**CLUSTER 2—Informed and collaborative discharge planning**			
21—Including rehabilitation therapists’ recommendations when considering discharge dates and location	4.30	4.26	4.28
2—Ensuring support (paid or unpaid) to meet patient needs when returning home	4.39	4.09	4.24
25—Patients and care partners being informed of what assistive devices are needed and where to get them	4.30	4.09	4.20
15—Having access to transitional spaces for those not yet ready to return home or waiting for a bed at their next point of care	4.22	4.13	4.18
61—Including home and community care providers in discharge planning	4.26	4.09	4.18
**CLUSTER 3—Access to transitional and outpatient services**			
64—Ensuring timely, consistent, and sufficient homecare services	4.39	4.26	4.33
60—Having access to necessary services and equipment regardless of ability to pay for it	4.35	4.17	4.26
**CLUSTER 4—Communication, education and knowledge acquisition**			
33—Providing patients and care partners with ongoing communication throughout their care transitions	4.52	4.26	4.39
73—Knowing what services exist and how to access them (e.g., homecare, community, social)	4.26	4.26	4.26
67—Addressing concerns and fears about going home	4.35	4.00	4.18
11—Having open conversations about what to expect about care and transitions	4.13	4.04	4.09
**CLUSTER 6—Person-centred care**			
5—Ensuring patients and care partners are included in decisions (e.g., goals, transition processes, and care plans)	4.61	4.35	4.48
37—Ensuring patients’ and care partners’ questions are answered	4.48	4.26	4.37
53—Using clear and simple language to improve patient and caregiver understanding	4.45	4.26	4.36
4—Respecting patients’ and Care partners’ preferences, knowledge, and needs	4.35	4.09	4.22
45—Discussing patients’ goals early and often throughout their care transitions	4.26	4.13	4.20
34—Communicating with patients and care partners in their primary language	4.22	4.09	4.16
58—Having a positive relationship with healthcare providers	4.26	4.00	4.13
74—Trusting that patients are receiving appropriate care that is in their best interest	4.09	4.13	4.11
10—Addressing concerns and fears about future falls	4.17	3.91	4.04
38—Being informed about medication changes (e.g., new, adjustments)	4.09	3.91	4.00
**CLUSTER 7—Physical, social, and cognitive activities and supports**			
12—Being compassionate while caring for patients	4.65	4.52	4.59
22—Encouraging patient motivation with rehabilitation	4.35	4.35	4.35
41—Ensuring patients feel supported physically and emotionally	4.39	4.22	4.31
27—Addressing delirium and cognitive impairment through best practices (e.g., visual aids, social interactions, routines)	4.39	4.17	4.28
29—Having individualized exercises that change over time to meet patient goals	4.39	4.04	4.22
40—Providing social, mental, and physical activities regardless of cognitive status	4.09	3.96	4.03
**CLUSTER 8—Provider knowledge, skills, roles, and behaviours**			
39—Having a care team that works well together	4.52	4.35	4.44
71—Having adequate pain management	4.45	4.30	4.38
31—Ensuring providers use the proper transferring techniques (e.g., sit to stand)	4.43	4.30	4.37
35—Ongoing patient assessments (e.g., geriatric, cognitive, functional) to identify, monitor, and manage clinical conditions	4.35	4.22	4.29
59—Having the necessary knowledge and skills to provide rehabilitative care for patients who have cognitive impairment	4.35	4.22	4.29
47—Practicing everyday activities (e.g., dressing, feeding, stairs, toileting) during recovery	4.22	4.13	4.18

Despite only 41 of the statements being in the go-zone, all statements were rated relatively high on both dimensions. The top statements rated most highly on importance and priority by participants included: *12—being compassionate while caring for patients* (mean = 4.65, 4.52; respectively); *5 –ensuring patients and caregivers are included in the decisions (e*.*g*., *goals*, *transition processes and care plans* (mean = 4.61, 4.35 respectively), *39 -Having a care team that works well together* (mean = 4.52, 4.35, respectively); *26—Having established partnerships between hospitals*, *rehabilitation facilities*, *and homecare services for consistent transitions* (mean = 4.43, 4.39, respectively); *33—Providing patients and care partners with ongoing communication throughout their care transitions* (mean = 4.52, 4.26, respectively); *71 –Having adequate pain management* (mean = 4.45, 4.30, respectively); *23 –Increasing staffing so providers can spend more time with patients* (mean = 4.48, 4.26, respectively); *37- Ensuring patients’ and care partners’ questions are answered* (mean = 4.48, 4.26, respectively); *20—Having access to different healthcare providers (e*.*g*., *geriatricians*, *therapists*, *social workers)* (mean = 4.43, 4.30, respectively); *31- Ensuring providers use the proper transferring techniques (e*.*g*., *sit to stand)* (mean = 4.43, 4.3, respectively); *53—Using clear and simple language to improve patient and caregiver understanding* (mean = 4.45, 4.26, respectively).

### Comparison of ratings between participant groups

PWLE and care partners generally rated the same clusters as being of greater importance and priority compared to healthcare providers and administrators; however, relatively small differences were noted in the magnitude of ratings. All clusters, except for *support for care partners*, were rated more highly on importance by patients and care partners than by healthcare providers and decision-makers. Patients and care partners rated all clusters more highly on priority than healthcare providers and decision-makers (see S5 Fig).

## Discussion

In this mixed-methods concept mapping study, we identified 74 statements that were rated on importance and priority to inform improvements in care transitions across the care journey for hip fracture. These statements were organized into eight clusters by participants. The majority of statements mapped onto concepts related to person-centred care and continuity of care. While most statements were rated highly on importance and priority, those specific to clinician knowledge, skills, roles, and behaviours were considered amongst the most important and highest priority statements.

One of the main findings from this study is that many statements mapped onto multiple interrelated aspects (clusters) of person-centred care and most were rated high on importance and priority. This implies an interconnectedness of the constituent requirements for effective care transitions. Well-established in the literature, person-centred care generally relates to the following areas of care: PWLE preferences, emotional support, physical comfort, information and education, continuity and transition, coordination of care, access to care, involvement of care partners [42, 43]. In addition to participants denoting ‘person-centred care’ as its own cluster, many other clusters also related to this approach, including statements relating to compassionate care, shared decision-making, and ongoing communication. By involving PWLE in decision-making, respecting their autonomy, and considering their preferences, person-centered care can promote a sense of empowerment and dignity throughout the transition process [43]; however, there is a need for more research in this area to examine the impact on patient-oriented outcomes [44, 45]. These person-centred principles are particularly important during vulnerable periods of care transitions, and as such, are recommended in quality standards on care transitions in the general population [46, 47].

Not surprisingly, many statements identified in our study also relate to Haggerty and colleagues’ conceptualization of continuity of care (often considered a dimension of person-centred care), which include relational (consistent persons involved in care fostering trust and familiarity), informational (use of information to link care from provider, setting or event), and management (organized care from several providers, adaptability to care needs) [48]. With respect to informational continuity, participants highlighted the importance of ensuring questions are answered, using clear and simple language, and providing ongoing communication throughout care transitions. Previous research on hip fracture care transitions has shown PWLE and care partners experience challenges with lack of information sharing and role confusion [25, 28, 30]. Asif and colleagues, in their scoping review, recommended leveraging written communication, patient navigator/peer support, and digital health technology as potential means to improve information sharing [25]. Similarly, Backman and colleagues’ recent scoping review [49] reviewed different digital health interventions in the literature for post hip fracture care. Most interventions identified focused on digital health technology to improve informational continuity [49], and included telehealth (virtual consultations), care transition/follow-up interventions (mobile and web-based applications with personalized management such as reminders, exercises, medication schedules), online education resources (videos and education modules on hip fractures, treatments, exercises and self-care), and wearable devices/sensor monitoring (e.g., clinicians can monitor remotely vital signs, activity). Overall, Backman and colleagues noted an absence of interventions related to other types of continuity, such as relational, as well as studies examining the acceptability, usability, and feasibility of using digital health technology to improve care continuity for this population. In our study, the use of technology to share educational materials about exercises and mobilization was rated as one of the least important and lowest priority statements. As such, for technology to be implemented and used to its full potential to improve care transitions for hip fracture, more work is needed to understand the unique needs of this population.

Findings in our study also highlighted the importance of management continuity, especially related to the concept of teams working well together and teams/persons working across healthcare sectors. For example, statement *26—Having established partnerships between hospitals*, *rehabilitation facilities*, *and homecare services for consistent transitions*, was rated high on importance and priority. Our findings support other research that has highlighted the potential benefits to integrated care pathways for hip fracture care [50–52]. In Ontario, bundled hip fracture care is currently recommended to support transitions between acute and rehabilitation hospitals [53]. A recent systematic review and meta-analysis reviewing randomized control trials (n = 12) showed integrated care for hip fractures improved activities of daily living at 6 and 12 months post-fracture [52]. In this review, integrated care was defined as “the cooperation of a multidisciplinary team including an orthopedic surgeon and geriatrician focused on elderly patients with hip fractures” (pg. 65). Notably, in this review, there was substantial variation of what constitutes integrated care (e.g., within hospital or across sectors), which is consistent with challenges reported in the literature [54, 55]. In our study, participants identified the importance of care pathways *across sectors* to be inclusive of, not only inpatient acute and inpatient rehabilitation, but also across community sectors such as primary care and homecare. Similarly, in efforts to improve integrated care in Ontario for populations with more complex health and social needs, the Ministry of Health has prioritized the formation of Ontario Health Teams to support care continuity across sectors, including primary care and community [56]. Given this focus on integrated care, there is likely a favorable environment to prioritize hip fracture care within these teams. Similarly, while there is a growing focus internationally on integrated care for improving continuity of care, patient outcomes, and experiences post-hip fracture [44, 57, 58], continued work is needed to understand the optimal composition of these teams and across what settings.

Extending beyond continuity, participants identified numerous statements related to clinician knowledge, skills, roles and behaviour, as they relate to hip fracture management (*71 –Having adequate pain management*, *27—Addressing delirium and cognitive impairment through best practices*, and *31 –Ensuring providers use the proper transferring techniques*). Sub-optimal acute pain management among those with hip fracture has been associated with increased risk of delirium, delayed mobility, longer hospital stays and poor outcomes (e.g., depression, sleep disturbances, chronic pain) [59]. Optimal hip fracture pain management often requires interdisciplinary collaboration, including medications as well as rehabilitation therapy [60]. The importance placed on clinical knowledge and skills has been supported by a recent scoping review by Djukanovic and colleagues in their examination of the meaning of care continuity among older adults [61]. Interestingly, Djukanovic identified that clinically skilled healthcare professionals were more important among older adults than having the same person involved in clinical care [61].

Moreover, when considering patient complexity, participants in our study emphasized the importance of ensuring adequate clinician knowledge, and skills to provide care for individuals with hip fracture who also experience cognitive impairment and delirium. In a scoping review conducted by Cadel and colleagues, 17 articles examining rehabilitation interventions for adults with hip fracture and cognitive impairment were identified [62]. The interventions were mostly focused on physical rehabilitation initiated in-hospital, with fewer interventions implemented across sectors. Of key importance, none of the studies qualitatively explored patient and care partner experiences with the interventions and specific to those with cognitive impairment and hip fracture, details on how to adapt the interventions to this population were lacking. As such, future research is warranted in exploring the unique barriers and opportunities to improve rehabilitation and care transitions for persons with hip fracture and cognitive impairment.

This study has several limitations to note. We experienced challenges recruiting patients and care partners. Recruitment occurred during the COVID-19 pandemic and our recruitment sites were impacted by capacity and resource restraints. However, there were no new relevant concepts being identified in the statements from the final PWLE and care partner brainstorming sessions. Healthcare providers in our study did not include any physicians, who may have had differing recommendations. Despite the intention to include physicians, we were unable to recruit any. Additionally, despite some diversity among healthcare providers and decision-makers, PWLE and care partners were all women and mostly white, reflecting a gap in our data from perspectives of those identifying as men and those from different ethnicities and language groups. Future research will be needed to capture more diverse perspectives, such as age, gender, race/ethnicity, language, and socio-economic status to inform the design of interventions to address diverse needs. Interestingly, while most of the participants were providers and decision-makers, findings still emphasized the importance of patient and care partners involvement in care and care transitions. This finding is notable, given that our study population consisted predominately of healthcare providers and decision-makers, who may be less likely to identify these necessary improvements than PWLE or care partner participants. Lastly, while this work identified actionable and prioritized statements for improving care transitions for individuals with hip fracture, future work must also assess the feasibility, acceptability, and appropriateness of developed interventions, prior to larger scalability.

Despite these limitations, there are several notable strengths of this study. Concept mapping is a highly participatory approach that puts participants at the centre of the research, giving them a voice in both data collection and analysis [35]. We included PWLE, care partners, healthcare providers, and decision-makers, which allowed us to gain a range of viewpoints, experiences, and perspectives related to recommendations to improve care transitions for adults with hip fracture.

## Conclusions

Seventy-four actionable recommendations mapped onto eights clusters to improve care transitions for individuals with hip fracture were identified in this study. Our results highlight the importance of meaningful patient and care partner engagement, with active involvement and a guiding role in all aspects of care including the transition process. Further, many statements included improvements in clinical knowledge and skills related to cognitive impairment, transfers, and pain management, emphasizing important areas for future work. This study provides insights for future interventions and quality improvement initiatives that focus on person-centredness, continuity of care, and clinician knowledge, skills, roles and behaviour to improve transitions in care among hip fracture populations.

## Supporting information

S1 Fig Point map. Each dot corresponds to a statement generated in the brainstorming sessions. Statement numbers are displayed in Table 2.
(TIF)

S2 Fig 7 and 8-cluster maps. Each shape represents a cluster containing statements (represented by dots). These maps were presented to participants in the mapping session.
(TIF)

S3 Fig Importance cluster rating map. The numbers of layers in a cluster represents its relative importance, with a greater number of layers being rated as more important.
(TIF)

S4 Fig Priority cluster rating map. The numbers of layers in a cluster represents its relative priority, with a greater number of layers being rated as a higher priority.
(TIF)

S5 Fig Pattern match diagram. The pattern match diagram illustrates how patients and care partners rated the clusters of statements on both dimensions (importance and priority) compared to healthcare providers and decision-makers. Abbreviations: PWLE–Persons with lived experience; HCP–healthcare providers; DM–decision-makers.
(TIF)
