# Supplementary figures and images for: Identifying and prioritizing recommendations to optimize transitions across the care journey for hip fractures: Results from a mixed-methods concept mapping study
Source: PLoS One. 2024 Aug 26;19(8):e0307769. doi: 10.1371/journal.pone.0307769 (PMC11346730; doi:10.1371/journal.pone.0307769)

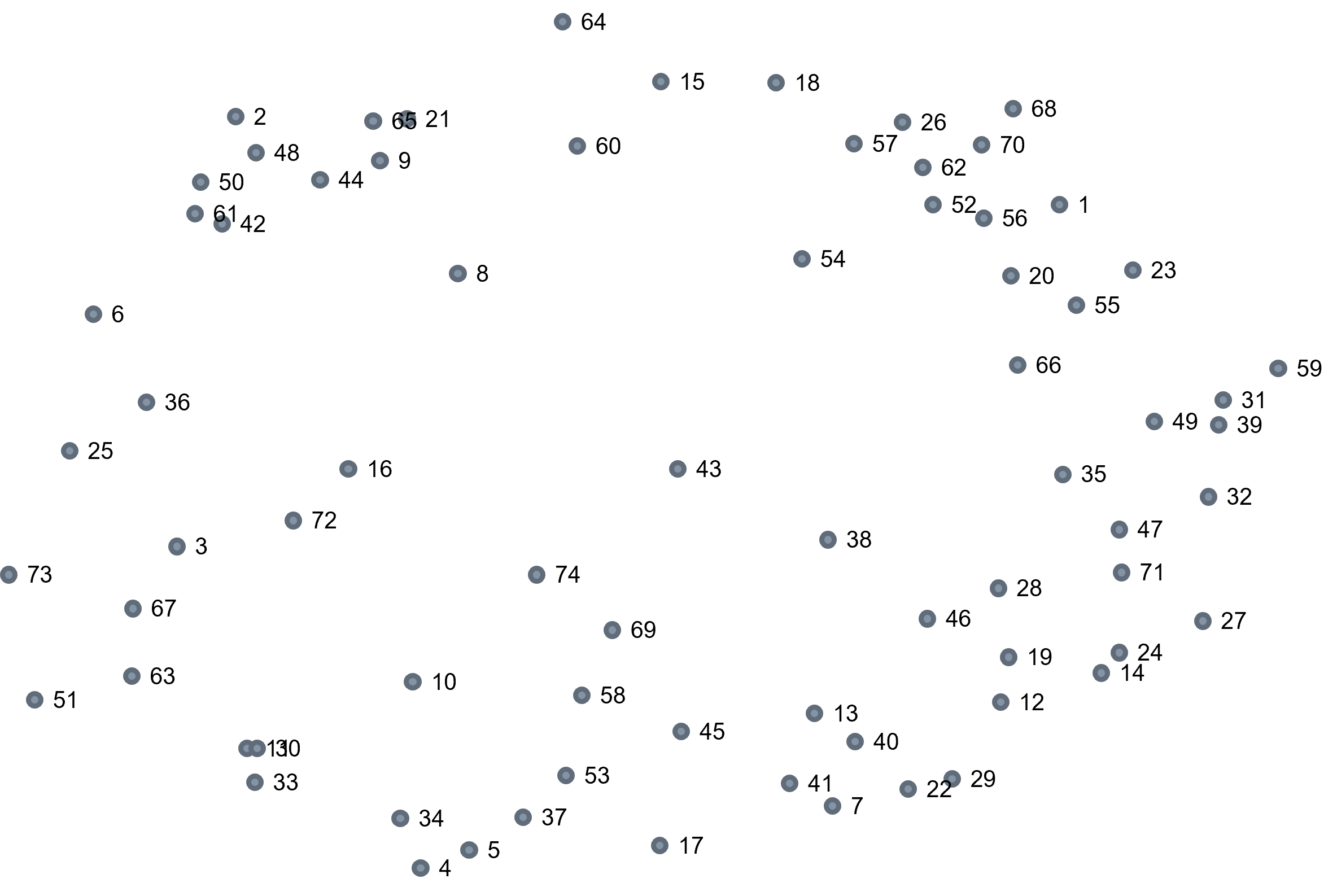

Supplement: S1 Fig — Each dot corresponds to a statement generated in the brainstorming sessions. Statement numbers are displayed in Table 2. (TIF) [file pone.0307769.s001.tif]

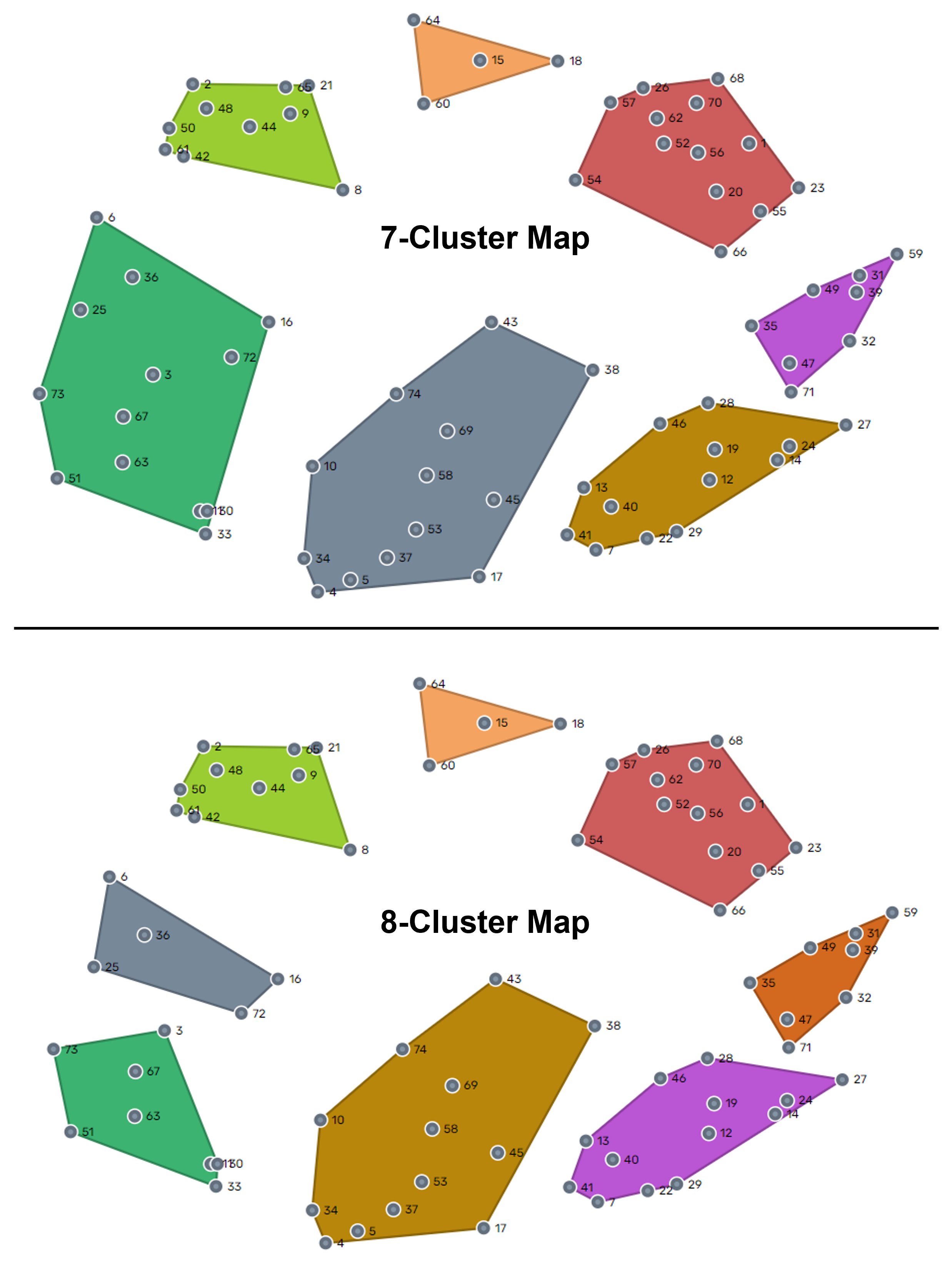

Supplement: S2 Fig — Each shape represents a cluster containing statements (represented by dots). These maps were presented to participants in the mapping session. (TIF) [file pone.0307769.s002.tif]

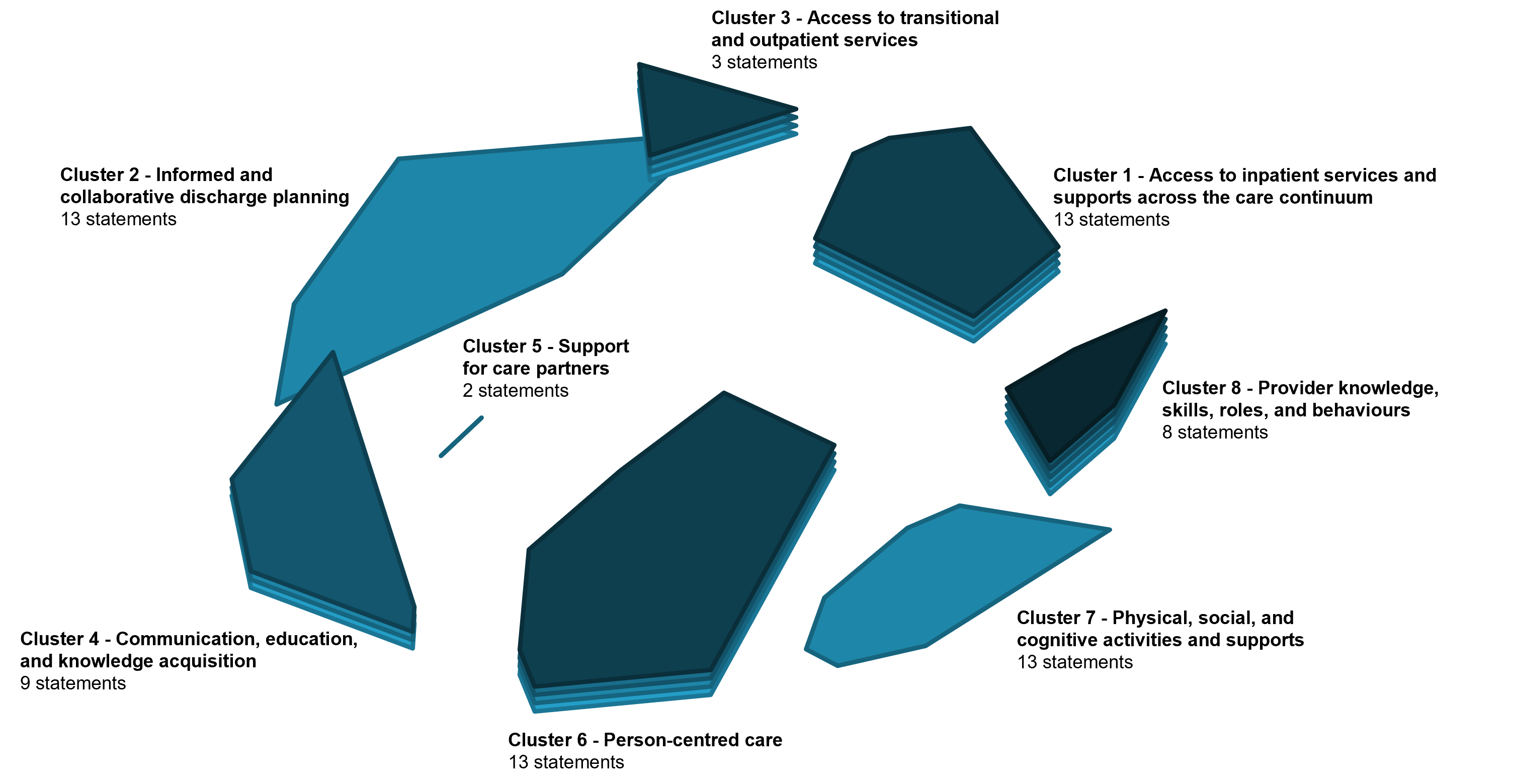

Supplement: S3 Fig — The numbers of layers in a cluster represents its relative importance, with a greater number of layers being rated as more important. (TIF) [file pone.0307769.s003.tif]

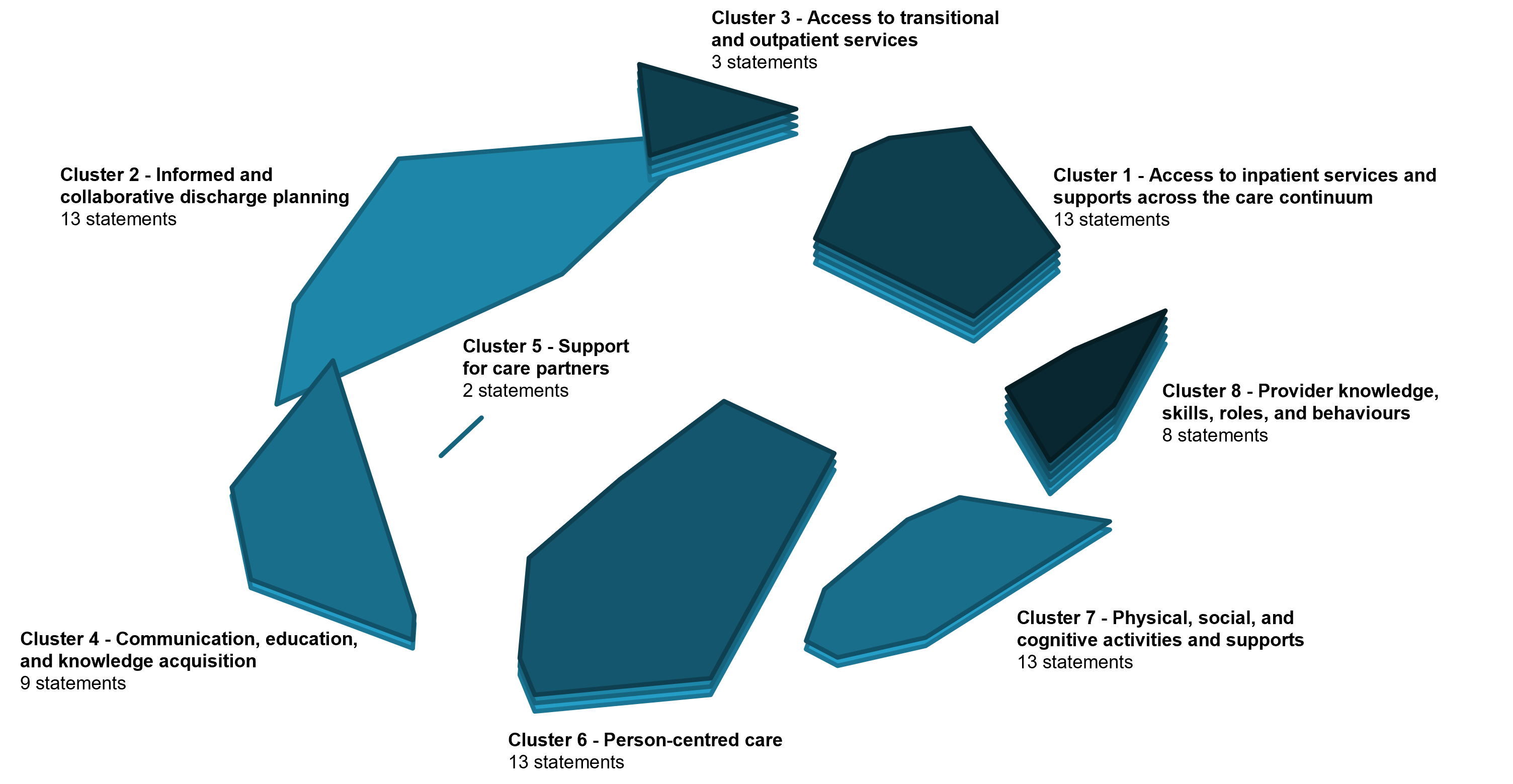

Supplement: S4 Fig — The numbers of layers in a cluster represents its relative priority, with a greater number of layers being rated as a higher priority. (TIF) [file pone.0307769.s004.tif]

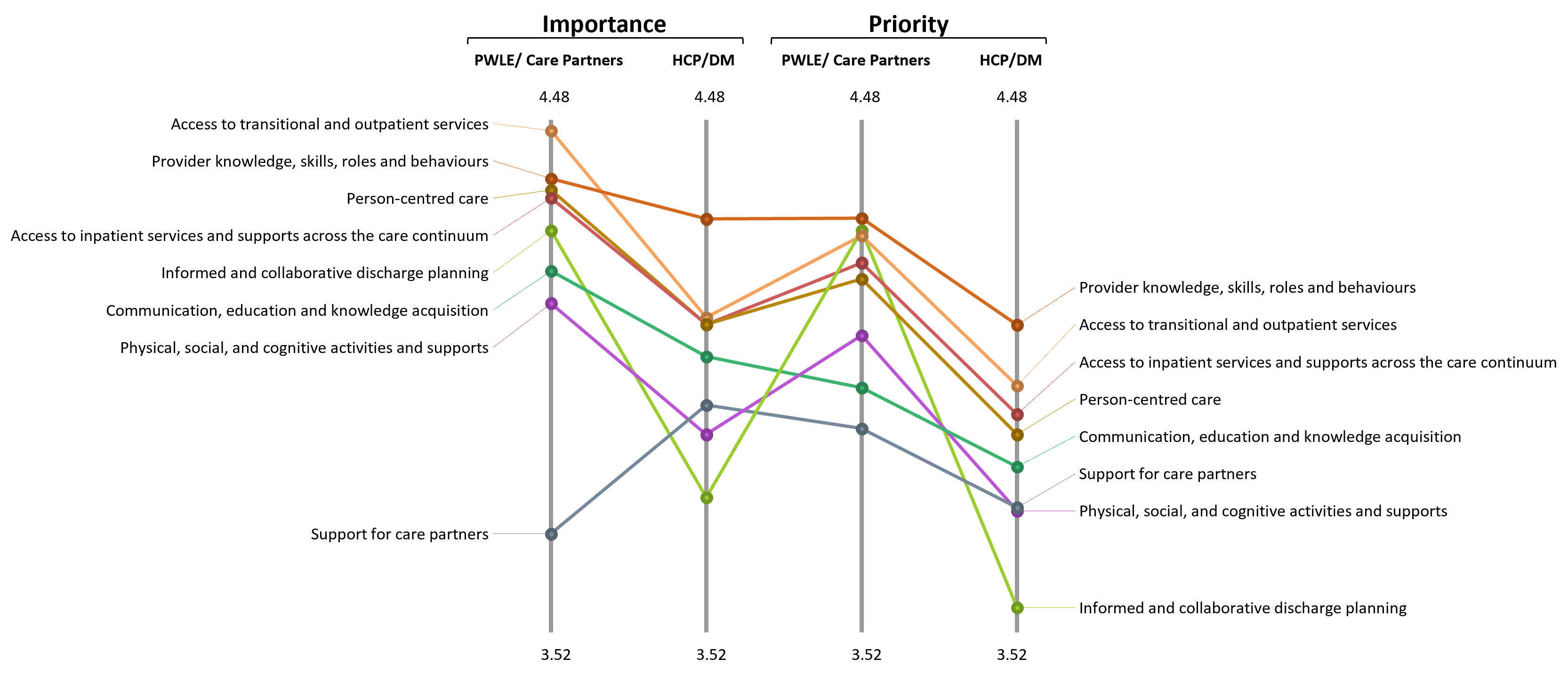

Supplement: S5 Fig — The pattern match diagram illustrates how patients and care partners rated the clusters of statements on both dimensions (importance and priority) compared to healthcare providers and decision-makers. Abbreviations: PWLE–Persons with lived experience; HCP–healthcare providers; DM–decision-makers. (TIF) [file pone.0307769.s005.tif]
